# Towards an Understanding of Retinal Diseases and Novel Treatment

**DOI:** 10.3390/ijms23147576

**Published:** 2022-07-08

**Authors:** Stephanie C. Joachim

**Affiliations:** Experimental Eye Research Institute, University Eye Hospital, Ruhr-University Bochum, In der Schornau 23-25, 44892 Bochum, Germany; stephanie-joachim@rub.de; Tel.: +49-234-2993156

The Special Issue, “Towards an Understanding of Retinal Diseases and Novel Treatment”, provides comprehensive information on retinal diseases such as glaucoma, age-related macular degeneration (AMD), diabetic retinopathy, retinitis pigmentosa (RP), and others. Globally, these diseases are, among uncorrected refractive errors and cataracts, leading causes of vision impairment. Especially in high-income countries, glaucoma, diabetic retinopathy, and AMD are, for example, more common reasons for vision impairment and blindness. The intention of this Special Issue is to provide insights into current research on these retinal diseases, which have complex pathomechanisms and are not fully understood yet.

Glaucoma is one of the leading causes of blindness worldwide, and the aging of our society will lead to a further increase in the number of people affected in the coming years. Vision loss occurs in patients due to the loss of retinal ganglion cells (RGCs) and their optic nerve axons. The main risk factor is increased intraocular pressure (IOP), leading to mechanical stress on the peripapillary sclera and the optic nerve head. However, the exact pathomechanisms of this disease are not yet understood. Among other factors, ischemic processes, excitotoxicity, and immunological mechanisms seem to be involved.

Decorin is a small leucine-rich proteoglycan and a natural inhibitor of several members of the transforming growth factor (TGF) family. This proteoglycan is present in the mouse optic nerve and is expressed by optic nerve astrocytes of mice as well as in the human optic nerve head. Decorin could attenuate the remodeling process in the optic nerve head in glaucoma and might be an interesting future therapeutic target [1]. TGF-β2 induced increased contractility, expression of α-smooth muscle actin, as well as an increased formation of actin stress fibers in human trabecular meshwork cells, which was mediated by connective tissue growth factor (CTGF). Since CTGF is upregulated in trabecular meshwork (TM) cells that are stressed, a new hypertension glaucoma mouse model was developed. Here, loss of RGCs is notable in 15-week-old mice. Weiss et al. observed that apoptotic processes precede neuronal death in this model. This was accompanied by a remodeling of synapses. The slow progression rate mimics the situation in glaucoma patients, thus making it a suitable model to study this disease more precisely [2]. Besides an elevated IOP, there are other factors contributing to glaucoma, such as an altered immune response. Benning et al. investigated the role of S100B in two different glaucoma models. Previous studies revealed glaucomatous neurodegeneration when applying S100B either systemically or intravitreally. Here, different synapse types were analyzed. While excitatory pre-synapses seem to be upregulated prior to the loss of RGCs, they are downregulated simultaneously with RGC death. Furthermore, excitatory post-synapses and RGCs degenerated at once. The results suggest a relevant role of synapse alterations as early events in glaucomatous degeneration [3].

Additionally, studies indicate an involvement of the WNT/β-catenin pathway, which, e.g., plays are role in cell proliferation and migration as well as apoptosis in glaucoma. The WNT/β-catenin pathway is considered to be involved in the pathophysiology of TM cells and might therefore affect IOP. Through activation of this pathway, cannabinoids could be an interesting therapeutic target for glaucoma in the future, affecting oxidative stress and inflammation [4].

In a glaucoma model, where high pressure was induced by ocular injection of Ad5.MYOC, pattern electroretinography revealed deficits, while RGCs loss did not occur yet. The authors identified changes in glial activation, inceptive neuroinflammation, activation of the complement system, and synaptic loss in the retinae of these animals via RNA sequencing [5].

The review by Al Hussein Al Awamlh et al. discusses if insulin signaling directly contributes to the pathogenesis of glaucoma or is rather a secondary response to the disease. There are trials for the use of intranasal insulin in other neurogenerative diseases. Due to the cross-talk insulin signaling and the various pathogenic events in glaucoma, which still need to be better understood, targeting insulin signaling might be a future therapy for glaucoma [6]. A further review on glaucoma by Mursch-Edlmayr and colleagues deals with the vascular aspects of glaucoma. There are emerging technologies to measure ocular perfusion to gain a better understanding of its role in this disease. Additionally, novel glaucoma drugs, e.g., Rho-kinase inhibitors, increase the outflow facility [7].

Glaucoma is also associated with glutamate-induced neurotoxicity. Intravitreal injection of N-methyl-D-aspartic acid (NMDA), a glutamate analog, leads to retinal degeneration in rats. Watanabe et al. investigated if this degeneration can be inhibited through metformin treatment. The study showed that metformin protected RGCs and amacrine cells against NMDA-induced excitotoxicity [8].

Muscarinic acetylcholine receptors, belonging to the superfamily of G-protein-coupled receptors, have five subtypes. The review by Ruan and colleagues describes the distribution and role of the individual subtypes in the retina. These receptors could be novel treatment targets, e.g., in glaucoma. The M1 receptor is involved in retinal neuron survival and might be a promising treatment target [9].

AMD is a progressive, neurodegenerative disease of the retina. The reason for the increasing vision loss is the accumulation of drusen between Bruch’s membrane and the retinal pigment epithelium (RPE). This results in undersupply (ischemia) and atrophy of the RPE, which ultimately leads to degeneration of the photoreceptor cells. Despite the knowledge gained so far, the exact pathogenesis of AMD is still unknown. Its complex pathology involves metabolic, functional, genetic, and environmental factors. Hypoxia, oxidative stress, and inflammation are among the contributing factors. Oxidative stress, an overproduction of reactive oxygen species, is involved in many neurodegenerative disorders, including retinal diseases such as AMD. Hence, antioxidants are an interesting treatment approach for AMD. In a study by Tosi et al., the possible anti-oxidative effect of N-acetyl-L-cysteine ethyl ester was analyzed in ARPE-19 cells and rats in comparison to N-acetyl-L-cysteine. In this study, N-acetyl-L-cysteine ethyl ester was more effective than N-acetyl-L-cysteine, e.g., it acted directly by reacting more rapidly with reactive oxygen species [10]. As mentioned, inflammation is a major pathomechanism in AMD. The RPE may contribute to retinal inflammation via activation of its Toll-like receptors (TLRs). The review by Klettner and Roider provides insights into the roles of TLRs in AMD, their activation in the RPE, and the implications of this for AMD pathophysiology. Especially the activation of TLR-2, -3, and -4 induces a profound pro-inflammatory response [11].

The WWC protein family is an upstream regulator of the Hippo signaling pathway, and WWC2 plays a crucial role during embryonic and postnatal angiogenesis. In their current study, Brucher and colleagues noted that WWC2 is an essential regulator of ocular angiogenesis in mice, while WWC1 might not be that important in this context [12].

With increasing age, lipofuscin accumulates in the RPE. The accumulation is mostly a result of the incomplete lysosomal digestion of photoreceptor outer segments, which contain high concentrations of docosahexaenoate. Products of docosahexaenoate oxidation exhibit photosensitizing properties, which can account for the photoreactivity of retinal lipofuscin [13].

Diabetic retinopathy (DR) commonly occurs in patients with diabetes. It begins with the early stage of mild non-proliferative DR (NPDR), which is defined by microaneurysms, retinal hemorrhage, intraretinal microvascular abnormalities, and changes in venous diameter. It then progresses to the proliferative DR (PDR) stage, which is characterized by pathological preretinal neovascularization. Additionally, in DR, inflammation is involved, leading to the production of cytokines, such as interleukin-17A (IL-17A). Since the aryl hydrocarbon receptor can halt the production of pro-inflammatory cytokines, enhance the proliferation of T regulatory cells, and suppress Th17 cell differentiation, its agonist VAF347 was investigated in diabetic mice. Systemic application of the agonist led to ablation of pathogenic Th17 cells and IL-17A, making it a therapeutic candidate for diabetes and DR [14].

In an in vitro blood–retinal barrier (BRB) model, based on human cells, treatment with high glucose in combination with the selective P2X7-receptor (P2X7R) agonist 2BzATP was investigated. This generated a BRB breakdown by enhancing barrier permeability. Increased expression of pro-inflammatory cytokines and P2X7R, vascular endothelial growth factor A (VEGF-A), and intercellular adhesion molecule (ICMA)-1 was noted as well as enhanced production of reactive oxygen species. The predicted P2X7R allosteric antagonist DHTS and the validated P2X7R antagonist JNJ47965567 significantly antagonized HG/BzATP-induced damage in this model [15].

The review by Iyer et al. discusses methods of vitreous sampling in DR. The authors describe major proteins and lipid mediators that were detected in diabetic vitreous samples [16].

Inherited retinal diseases are rare disorders caused by genetic anomalies. They include RP, Leber congenital amaurosis, early onset retinal dystrophy, and Usher syndrome. The RP leads to dystrophy of the photoreceptors. A novel mutation in gene PRF1 that causes RP could be identified using family-based whole-exome sequencing. In a Taiwanese family, six snps candidates were identified as RP disease-causing variants, one the novel pathogenic variant PRF1 [17]. Variants in the RPE65 gene account for 1–6% of RP and 3–16% of Leber congenital amaurosis and early onset severe retinal dystrophy cases. There is now a gene therapy for the treatment of RPE65-based inherited retinal diseases available. Hence, testing patients is crucial to find out if this treatment would be helpful. The review by Aoun et al. illustrates molecular testing possibilities of patients that represent possible candidates for the RPE65-gene supplementation therapy [18]. In addition, the review article by Chiu et al. deals with current developments in gene therapy for inherited retinal dystrophies, especially RPE65-gene therapy for Leber congenital amaurosis [19].

Several animal models are available to study RP, but there is still a lack of human-representative animal models. In a new rat model for RP, a knockout at lecithin retinol acyltransferase (LRAT) is based on a patient group harboring a homologous homozygous frameshift mutation in the LRAT gene. Visual examination and further evaluations, such as optical coherence tomography measurements and electroretinography, showed characteristics of RP without debris in the subretinal space making this an interesting new model to study RP [20].

All original articles and reviews of this Special Issue deal with various retinal diseases to contribute to a better understanding of pathogenesis and novel treatment options.
